# Identification of Parameters That Predict Sport Climbing Performance

**DOI:** 10.3389/fpsyg.2019.01294

**Published:** 2019-05-31

**Authors:** Xavier Sanchez, M. Torregrossa, T. Woodman, G. Jones, D. J. Llewellyn

**Affiliations:** ^1^Department of Health and Sport, Halmstad University, Halmstad, Sweden; ^2^Department of Psychology, Autonomous University of Barcelona, Barcelona, Spain; ^3^School of Sport, Health and Exercise Sciences, Bangor University, Bangor, United Kingdom; ^4^School of Clinical and Applied Sciences, Leeds Beckett University, Leeds, United Kingdom; ^5^Medial School, University of Exeter, Exeter, United Kingdom

**Keywords:** climbing, extreme sports, lead-climbing, performance parameters, qualitative analysis, route finding, route previewing

## Abstract

In recent years, extreme sport-related pursuits including climbing have emerged not only as recreational activities but as competitive sports. Today, sport climbing is a rapidly developing, competitive sport included in the 2020 Olympic Games official program. Given recent developments, the understanding of which factors may influence actual climbing performance becomes critical. The present study aimed at identifying key performance parameters as perceived by experts in predicting actual lead sport climbing performance. Ten male (*M*_age_ = 28, *SD* = 6.6 years) expert climbers (7a+ to 8b on-sight French Rating Scale of Difficulty), who were also registered as climbing coaches, participated in semi-structured interviews. Participants’ responses were subjected to inductive-deductive content analysis. Several performance parameters were identified: passing cruxes, strength and conditioning aspects, interaction with the environment, possessing a good climbing movement repertoire, risk management, route management, mental balance, peer communication, and route preview. Route previewing emerged as critical when it comes to preparing and planning ascents, both cognitively and physically. That is, when optimizing decision making in relation to progressing on the route (ascent strategy forecasting) and when enhancing strategic management in relation to the effort exerted on the route (ascent effort forecasting). Participants described how such planning for the ascent allows them to: select an accurate and comprehensive movement repertoire relative to the specific demands of the route and reject ineffective movements; optimize effective movements; and link different movements upward. As the sport of climbing continues to develop, our findings provide a basis for further research that shall examine further how, each of these performance parameters identified, can most effectively be enhanced and optimized to influence performance positively. In addition, the present study provides a comprehensive view of parameters to consider when planning, designing and delivering holistic and coherent training programs aimed at enhancing climbing performance.

## Introduction

In recent years, extreme sport-related pursuits such as climbing not only have emerged as recreational activities but have evolved into competitive mainstream sporting disciplines (Mittelstaedt, 1997; Hoibian, 2017). Next to growing practice of sport climbing as a leisure activity, competition environments and practices have been developing internationally since the first World Championships held in Germany in 1991. Today, the sport of climbing is a discipline included in the 2020 Olympic Games official program in Tokyo, and it is proposed for inclusion in the 2024 Olympic Games official program in Paris. Despite the systematic and intensified training of athletes, the identification and understanding of key factors that may influence actual sport climbing performance is warranted. Therefore, the present study aimed at identifying key performance parameters as perceived by experts in predicting actual lead sport climbing performance.

Sport climbing takes place indoors as well as outdoors, and utilizes a belayed dynamic rope, which is attached to the climber. The belayed rope is connected to pre-fixed anchor points during the ascent by the climber and the system acts to protect the climber in the event of a fall. From a sporting point of view (see Stiehl and Ramsey, 2005), climbing is unique because of the different vertical practice environments. Climbers are routinely required to ascend and/or descend and/or traverse a given surface to complete the route. Sport lead climbing in particular may be performed indoor or outdoor; indoor on an artificial surface using artificial holds and outdoor on a variety of natural rock types and surfaces. The range of different types of climbing behavior that exist and the number of permutations of such behaviors makes categorizing individual climbing activity challenging [see taxonomy and further discussion in Jones and Johnson (2016)].

The emergence of climbing as a competitive discipline has generated research interest. Studies have mainly examined motivational and risk-taking profiles (e.g., Martha et al., 2009; Jones et al., 2017), physiological aspects (e.g., Espanña-Romero et al., 2012), biomechanical properties (e.g., Vigouroux et al., 2011), and injuries (e.g., Jones et al., 2018). Notwithstanding previous work, researchers frequently discuss that variables of a psychological nature such as problem-solving ability, movement sequence recall, route finding skills, anxiety levels and stress management may be better predictors of optimal physical climbing performance than physiological or biomechanical variables (e.g., Sheel, 2004; Watts, 2004; Giles et al., 2006; MacLeod et al., 2007; Morrison and Schöffl, 2007; Jones and Sanchez, 2017).

However, psychology-based research has rather utilized climbing, typically, as a task to fulfill its experimental designs and reach its research goals by adapting climbing rules and adjusting climbing routes (e.g., Smyth and Waller, 1998; Boschker et al., 2002; Pijpers et al., 2005). In sport climbing in general and in climbing competition in particular most of these *modifications*, if not all, are not permitted. In addition, whilst previous studies within other areas of sport sciences had recruited elite/expert climbers as well as novices, most psychology-based research had basically recruited inexperienced/novice participants only. The main objective of the present study was to identify sport climbing performance predictors so that future research can, ultimately, influence both practice and performance.

In the sport of climbing, the consequence of a fall whilst leading is complex and largely determined by the ability of the belayer to arrest the fall, the length of fall, non-failure of the protective equipment and whether impact is made with the climbing surface during or at the termination of the fall. Climbers may elect to pre-practice a route prior to leading. Pre-practice allows climbers to rehearse movement sequences, evaluate potential risks and the likelihood of success in relative safety. It is common for individuals preparing to lead outdoor routes of the highest standards to pre-practice the ascent thereby increasing the chance of success and reduce the chance of failure due to a fall. Ascending a climbing route without conducting pre-practice is considered in the arena to be the personification of climbing performance. Critically, physical pre-practice is not permitted in competition – performances are what are labeled as “on-sight” – but a standardized time to visually preview the route prior to ascent by participants is allowed (i.e., route previewing). Little research has examined the psychological requirements of rock climbing although they are thought to be “a key element in accomplished climbers” (Morrison and Schöffl, 2007, p. 852).

Route previewing is one of such variables suggested by sport scientists as a determinant to successful climbing performance (e.g., MacLeod et al., 2007; Sanchez et al., 2012; Seifert et al., 2017). In the same line, sport psychology researchers have suggested that route previewing mistakes are “a major reason for falling during climbing” (Boschker et al., 2002, p. 25) and elite climbing competitors have reported that a lack of climbing route knowledge is a handicap prior to performing in competition (Ferrand et al., 2006). In climbing, when performing on-sight, mistakes are not allowed: falling off the wall is not only the end of that given ascent; it also means for the climber that the given route will have never been performed on-sight, ever. Therefore, a second objective of the present study was to describe the role and function of route previewing, understood as the pre-ascent visual inspection of the climbing route, which past research had suggested as a climbing-specific, psychological-in-nature variable for successful performance (Ferrand et al., 2006; MacLeod et al., 2007; Sanchez et al., 2012; Seifert et al., 2017).

Given the current limited understanding of the factors that influence climbing performance, in the present study we adopted Bishop’s (2008) first stage of his sport sciences applied research model for sport sciences (ARMSS). Precisely, ARMSS is a three-phase, eight-stage model, originally proposed for injury prevention and health research (Finch, 2006; Sussman et al., 2006), which provides a framework of particular interest to such *new* sporting disciplines for which performance predictors are still to be identified. These three phases evolve from description to experimentation and implementation with the following different stages being proposed within (see Bishop, 2008, for full details). Bishop’s (2008) ARMSS first stage highlights the need for discussion with athletes and coaches to gain a thorough understanding of the sport and its performance-related issues before proceeding to the following stages of the model. Therefore, we applied a qualitative analytic approach (Patton, 2002) to gain a comprehensive understanding of the key performance parameters as perceived by experts in predicting actual sport lead-climbing performance.

## Materials and Methods

### Participants

A purposive sample was adopted (Berg, 2001; Patton, 2002) to represent expert climbers with coaching climbing badges; inclusion criteria included (a) domain-specific indicators of personal climbing performance and (b) officially regulated awards that indicated coaching education in sport climbing. On the one side, interviewees were required to possess an advanced to elite current climbing ability level superior to an on-sight 7a French Rating Scale of Difficulty [F-RSD; see Draper et al. (2016) for comparative amongst climbing rating systems]. Current ability rather than climbing experience was gathered as a measure of expertise since an individual’s climbing standard can vary throughout a single year. Research has categorized climbers possessing an ability superior to 7a F-RSD as advanced performers (Draper et al., 2016), as individuals require excellent skills, strength and time commitment to maintain such a standard ability level (Cox and Fulsaas, 2003). On the other side, interviewees were also required to hold, at least, a national officially recognized coaching qualification in sport climbing. Given the purpose of the study, it was deemed significant to interview climbers who, in addition to being experts in the practice of climbing would also possess an officially recognized education in teaching and coaching climbing, and thus who would be used to provide feedback, incorporate prompts, correct and reinstruct, use questioning and clarifying, and engage in instruction (Douge and Hastie, 1993).

The final sample interviewed in the present study comprised 10 male climbers (*M*_age_ = 28, *SD* = 6.6 years, range 21–40) with an on-sight climbing ability ranging from 7a+ to 8b F-RSD (advanced to elite) and a climbing experience ranging from 8 to 22 years of practice (*M*_exp_ = 14, *SD* = 6.2 years). In addition, four participants were in possession of the Royal Belgian Alpinism Federation (*Royale Club Alpin Belge*) qualification whereas the other six possessed the Belgian Physical Education and Sport (*ADministration de l’Education Physique et du Sport*) qualification. Amongst the ten participants, six also held a Higher Education taught degree in physical education. Participants practiced in a wide range of other sports including ice-climbing, sky-diving, paragliding, swimming, gymnastics, and cycling.

### The Interview Guide

Two psychologists were first involved in the construction of the semi-structured interview guide. The open-ended questions were generated based on existing climbing-specific literature (Deweze and Le Menestrel, 1987; Salomon and Vigier, 1989; Glowacz and Pohl, 1992; Lemare and Morrot, 1995; Sheel, 2004; Watts, 2004; Giles et al., 2006). As per previous research (e.g., Holt and Morley, 2004; Wolfenden and Holt, 2005), the main topics of the interview guide were predetermined (deductive process) but the different questions were open-ended to elicit a range of responses pertinent to, and generated by, each participant (inductive process).

Pilot interviews were conducted with two participants matching the pre-established study participation criteria. These two climber-coaches were interviewed individually and were not included in the final sample. A second researcher, who acted as a passive observer, was present during the interviews. After each pilot interview, the two researchers and the interviewee discussed the interview with a view to (a) enhance interviewing procedures and interviewer skills and (b) refine the phraseology and technical jargon employed by the interviewer.

The final interview guide consisted of four main sections. The first section gathered general demographic information such as age, occupation, educational and coaching backgrounds, and sports practice. Also, specific sport climbing information such as reasons, frequency and types of practice were gathered. In the second and third sections, questions revolved specifically around the participants’ experience of “on-sight” (section “The Interview Guide”) and “pre-practiced” (section “The Interview Guide”) climbing modalities in lead sport climbing. An “on-sight” ascent is characterized by time-constraint visual examination of the route before climbing without previous physical rehearsal, and then ascent without a single fall. One may attempt an on-sight climb and fail the challenge. Subsequent successful attempts on that same route are considered “pre-practiced” ascents (a climb on a physically pre-rehearsed route). Examples of questions in these two sections were: “what do you do to prepare for an on-sight/pre-practiced climb and how do you do it?”; “what do you focus on when climbing on-sight/pre-practiced?”; “do you train with a view to climb on-sight/pre-practiced?”; “how do you prepare/work for particularly difficult parts of the route (e.g., cruxes)?” Discussion with the participants in these sections revolved around the factors that they believed influence climbing performance. The last section of the interview guide (section 4) focused particularly on route previewing in sport climbing. Participants were first asked to define, in their own words, the concept of “route preview/previewing” and then were asked to discuss its role and functions, both when climbing on-sight and pre-practiced. The complete interview guide is available from the corresponding author.

### Procedures

Climbers were individually approached at five different indoor climbing facilities across the French-speaking part of Belgium and invited to take part in this qualitative study investigating climbing performance. Note that contact with these facilities was regular during a period of time within the scope of a wider project that examined psychological aspects of sport climbing in a series of different studies (Sanchez and Dauby, 2009; Sanchez et al., 2010, 2012). All participants received written and verbal information about the study and were informed that they could withdraw from the study at any time. Confidentiality was assured by giving each interviewee a participant number, which was used as the only identification code (e.g., P1). The investigators ensured that the participants understood the overall purpose of the present qualitative study.

The interviews were carried out by the first investigator, who had both experience working with international elite athletes and coaches as a psychologist, and had experience of indoor sport climbing. All these aspects facilitated communication with the participants during the meetings and ensured rigor from the investigator during the course of the research. All interviews were conducted face-to-face, individually, either in a meeting room at the participant’s climbing club or in a laboratory at the interviewer’s university. All interviews were recorded using a dictaphone and their duration ranged from 65 to 110 min.

Each interview began by reminding the participants the nature and purpose of the study. Then, the different sections of the interview guide were followed in the pre-established order. Although the same questions were asked to all participants in a similar manner, the order of the questions within each section was allowed to vary according to the flow of the interaction between the interviewer and the interviewee. The interviewer allowed the participants to express themselves freely, to enhance the richness of the information (Patton, 2002).

In addition, consistent with the semi-structured interview format and use of open-ended questions (Patton, 2002), probes were asked throughout the interview, “to elicit more information about whatever the respondent has already said in response to a question” (Berg, 2001, p. 76). This encouraged the climbers to expand on their answers (elaboration) and allowed a more thorough understanding of their responses (clarification) (Rubin and Rubin, 2005). This knowledge elicitation took place using Spradley’s (1979) three types of questions for interviewing: (1) “descriptive questions” to identify precisely what was being discussed (e.g., “Can you describe what you do to prepare yourself for an on-sight climb? Can you tell me which are, in your opinion, the key aspects for success when climbing pre-practiced?”); (2) “structural questions” to obtain as much information as possible about the different aspects mentioned (e.g., “You mentioned that resting points and cruxes are crucial parts of a route. Why are they so important? What do you do to find them? What do you do to optimally face/utilize them?”); and (3) “contrast questions” to clarify and distinguish what the informant meant by the various terms and situations used (e.g., “Which are the differences between climbing a route on-sight or pre-practiced? Does it change to face a crux at the beginning of the route compared to facing it at the end of that route?”). These data collection procedures were adopted to elicit relevant knowledge from the sample interviewed. The same approach was used for each interview.

### Data Analysis

Interviews were transcribed verbatim, yielding 130 pages of single-spaced typed text. All participants were given the opportunity to check their own interview transcript for content accuracy and validity. Most participants (6 out of 10) checked and returned their interview transcript; no significant changes to the transcripts were noted. The transcriptions of the interviews, which were defined as a hermeneutic unit, were content analyzed using ATLAS/ti qualitative data analysis software package (Muhr, 1997). ATLAS/ti is a flexible tool for constructing networks, particularly suited for approaches of theory building (see Kelle, 1995 for an overview on qualitative methodology and computer software).

A hierarchical content analysis was carried out by the first and second authors on the textual information to classify and reduce the data to more relevant and manageable units (Sparkes and Smith, 2014). The analysis of the transcripts, which departed from raw data, kept as a reference the interview guide and alternated an inductive category development -bottom-up or data-driven- with a deductive category application -top-down or theory-driven (Patton, 2002). Inter-rater reliability amongst two first authors aimed at ensuring that all units of meaning, themes and categories emerging from the information provided by the climbers were appropriately created, grouped and categorized (Sparkes and Smith, 2014). Member checking took place for trustworthiness (Smith and McGannon, 2018); findings were verified by the participants who agreed to do so (*n* = 6) once analysis procedures were finalized.

## Results

From the content analysis, several performance parameters perceived by the climbers as most influential emerged: passing cruxes, strength and conditioning aspects, interaction with the environment, possessing a good climbing movement repertoire, risk management, route management, mental balance, peer communication, and route preview. Route previewing emerged as critical when it comes to preparing and planning ascents, both cognitively and physically. That is, when optimizing decision making in relation to progressing on the route (ascent strategy forecasting) and when enhancing strategic management in relation to the effort exerted on the route (ascent effort forecasting). Table 1 provides their frequencies, per participant and overall. A sample of participants’ quotes for each climbing performance predictor is provided in Table 2.

**Table 1:** Coded performance predictors frequencies (per participant and totals).

	P 1	P 2	P 3	P 4	P 5	P 6	P 7	P 8	P 9	P 10	Total
Mental balance	1	1	0	0	0	0	0	0	0	0	2
Peer communication	1	1	1	1	1	1	2	0	1	0	9
Optimal route previewing	1	2	0	6	4	0	2	2	2	3	22
Training in route previewing	2	3	5	4	1	1	1	1	1	1	20
Passing cruxes	1	1	0	0	3	2	4	4	3	2	20
Physical aspects: strength and conditioning	5	1	2	1	2	2	2	0	5	0	20
Interaction with environment	0	0	3	2	0	2	0	0	3	1	11
Climbing movements repertoire	7	1	1	3	0	5	1	0	3	0	21
Risk management	2	0	0	0	1	0	0	0	2	1	6
Route management	6	1	2	2	4	0	0	0	0	0	15
Total	26	11	14	19	16	13	12	7	20	8	


**Table 2:** Sample of participants’ quotes for each perceived predictor of sport climbing performance.

Mental balance	**P2:** Based on belief, you build up or copy habits because you believe they will have an influence on your performance… sleep, eating habits… with the goal of achieving top performance.
Peer communication	**P1:** To improve myself and make progress in climbing, I used to talk and discuss everything with my friends… from how to do it, to what to change to improve things, etc. And that’s what I try to do in my work now as trainer… **P3:** Talking things over with people is obviously necessary to come up with the optimal method…
Optimal route previewing	**P4:** The quality of the route previewing is a determining factor: poor route finding leads to poorer performance. **P9:** Since I’m not interested in performance, sometimes I don’t take time to preview the route and just set off and try it… but if you’re interested in performance, you have to be patient and do more systematic, methodical route previewing…
Training in route previewing	**P3:** It’s obvious that it’s important to train yourself in how to preview routes if you’re going after performance! **P10:** With people I train, I teach them route finding, for example by having two people preview the same route together and you see if they are ineffective and why they are ineffective. But generally speaking it just boils down to being interested… you have to see it as a challenge or else you just go off on a route, like that…
Passing cruxes	**P5:** When my aim is performance, I concentrate on the hard parts… It’s important to aim for the cruxes… The easy places: they take care of themselves… But the difficult ones, the cruxes, that’s something else again: I try to memorize them (especially the distance/length of the crux, and what they are like: if there’s some special characteristic about it) and to picture them in my head. That way, when I reach a certain section of the route, I know where and how to position myself (hands and feet… that’s it, more or less!)…
Physical aspects: strength and conditioning	**P2:** To reach the best level possible you’ve got to work on the wall, to train specific muscles... **P9:** The size and body type of the climber plays an important role!!!... That’s how it is: two climbers with the same physical-body type will generally end up spotting the same difficulties when previewing the route…
Interaction with environment	**P3:** A “general pattern” exists, but every climber (style, technique, strength, body shape) adapts this model to his own characteristics. But this adaptation will also depend on the level of the route: the higher the level of the route is, the less choice there will be sorting it out! And this is even more so in the hall. **P4:** Relative to a given route, top performance depends on the interaction between the climber and the characteristics of the route to be climbed.
Climbing movements repertoire	**P4:** I work a lot (route finding and afterward performance) by comparing the movement to be done with the ones in our repertoire (knowledge base, I suppose you’d say). It’s very possible to find sequences that are more or less the same (or moves) for different routes. The stock of movements (repertoire) is key in climbing. **P6:** The fact is, there is a certain range of climbing movements that you can teach people you train, but it’s always the same movements you’d come up with anyway… the only thing that changes according to the size of the hold is the orientation of the wall, etc.… but a crux will always be a crux! So like with the feel of the crux you have, then you know you’ve already been on a wall incline just like the one you’re reading, so you know you’ve got all that same stuff to do… it’s always comparing what you know and what you see!
Risk management	**P9:** The ideal way to use a route or pass (definition) is the one that’s the most efficient and where you’ll be running the least risk of falling! Because it’s also important not to take an unnecessary risk, so you don’t fall… keeping in mind that risk is an objective thing… **P10:** Between a route that will take lots of my energy but where I’m sure I’ll succeed (and not fall), and a route that will take less of my energy but where the risk of falling is higher, I choose the most efficient one (less energy without falling!). What I mean is first and foremost the one that’s the safest… because falling is the end of the route! Now, if the risk is great but I’m not going to fall, then that will depend on how the route is and afterward will depend on where I am on the route… if it’s the end of the route than I can let myself take a risk, ok… that’s a possibility… but if it’s at the beginning then I’m not going to take any risks… if it’s at the end, with my kind of climbing style, my energy is sure to be at its limit anyway, and so to waste a lot of energy would be asking for a fall, so… either things go easy or I’ll get tired and then I’ll fall anyway!
Route management	**P8:** Climbing a route badly doesn’t just mean falling… not just that!!! I can reach the top of a route and do it all over again because I was too tense… climbing badly is being choppy, using much too much energy for the effort the route demands… and more than anything, not getting any fun out of the route and its moves. You can see when someone “climbs a route badly” if it’s choppy, if someone is tense with his grips… you see it right off… which doesn’t mean mixing up fast-dynamic with tense!


**FIGURE 1:**
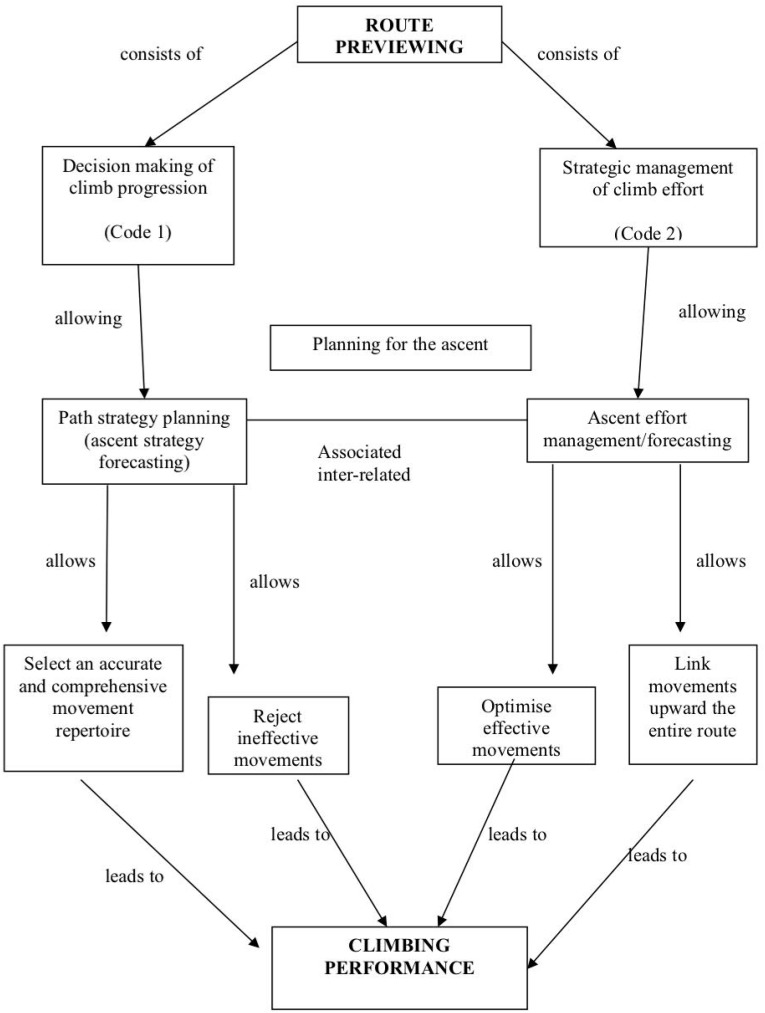
Perceived role and function of route previewing in sport climbing performance.

Moreover, participants described how such planning for the ascent allows them to: select an accurate and comprehensive movement repertoire relative to the specific demands of the route and reject ineffective movements; optimize effective movements; and link different movements upward. Our sample of expert and advanced climbers believed that these two aspects, that is path strategy planning (ascent strategy forecasting) and ascent effort management were inter-related (see Figure 1).

More precisely, route previewing would help climbers to acquire an accurate and comprehensive movement repertoire, to eliminate ineffective movements and optimize the effective ones, and to determine how to link these movements upward, over the entire route. Our sample considered route finding necessary for both the mental preparation of a route never climbed before (on-sight performance, competition) and the actual practice of cruxes in training sessions (training, learning). Climbers noted that performance outputs were, ultimately, determined by both the factors contributing to the climber’s profile (e.g., anthropometric characteristics) and the route itself (e.g., traverse, overhanging). Table 3 provides their frequencies, per participant and overall. A sample of participants’ quotes for the perceived role and functions of route previewing is provided in Table 4.

**Table 3:** Coded route previewing-related frequencies (per participant and totals).

	P1	P2	P3	P4	P5	P6	P7	P8	P9	P10	Total
Decision making of climb progression^1^	18	7	3	1	4	9	9	6	11	6	74
Strategic management of climb effort^2^	1	1	1	2	5	5	4	2	7	9	37
Total	19	8	4	3	9	14	13	8	18	15	


*^*1*^Code 1 in Figure 1. ^*2*^Code 2 in Figure 1.*

**Table 4:** Participants’ quotes sample for the perceived role and function of route previewing in sport climbing performance.

Decision-making of climb progression	**P1:** What I try to do, to teach them every time, is to spot the hard sections and the easy sections, to intersperse the rest position points and the non-rest positions… Afterward, if we have the time, we’ll try to spot more specific moves (but sometimes, like when competing, there’s no time to do all that). Spot instead. The “left-right-left” so you don’t do it wrong! The hidden holds… And especially spot the points or the different parts of the route: “there it’s hard, watch out there because there’s no resting point, over there it’s easier…”
	**P4:** The ideal thing is to see the sequence as a whole (feet and hands): sequence by sequence, the series of moves (like a collection of photos). Route finding steps: This is all about a process going from broader and general to more specific and fine-tuned: (1) Look at the route as a whole: (a) Trace it out (without focusing). You need to understand the route so you can focus afterward; (b) With as wide a scope as possible (like a movie). (2) Memorize the route: (a) Zoom in to spot the details; (b) Get the plan as firmed up as possible. (3) Read in sequences: (a) Figure out the various ways of succeeding; (b) Choose the moves best suited for each one…
	**P7:** Actually, speed in making the moves is a factor in success… a result of correct route finding among other things… in fact, two climbers at the same level can have different levels of route finding. You can see this because “the best finder” will bind the sections together faster (very fast), for example, he’ll be stable in every position (or almost every one), he’ll take the best advantage of the resting points, manage the route and the transitions between the parts correctly, the easier sections, etc. And from there you get a whole efficient climbing body-language strategy, which goes for the moves as well… you see a fluid series, fine-tuned moves, etc.
	**P8:** When I’m reading the route from the ground, instead of thinking about how I’m going to crimp the hold (because often it’s hard to see its exact characteristics, maybe it’s blocked and so I can’t crimp it the way I planned from the ground), I spot how I’m going to use it in the best way. What I’ll do when I’m there (rest, dyno, etc.) and see what’s got to be done, what’s going to happen next… now, if I know how I’m going to do it, that’s just great, but my first concern is to know what I’m going to do when I’m there, so I don’t waste time!

Strategic management of climb effort/exertion	**P2:** The basic principle of climbing is to save your arms by pulling yourself up by your feet…
	**P3:** Hold back… as soon as you can, you have to rest. It’s important to spot all the resting points on the route as far as possible, especially visually, and to use them! Find the most efficient path you can: this is one of the key tips you should never forget. Listen to your body and feel it: when you feel tired, it’s already too late… too much lactic acid in the muscles!
	**P4:** The goal is to find the most efficient movement, the fastest one possible (the slower you go, the less your chances of succeeding).
	**P5:** This is a climbing basic: be as efficient as you can! When you’re reading, you have to see what hold to take further on before taking the one right before that one… while taking great care to save energy… and sometimes there are holds that don’t get you anywhere… or on the other hand, you find there is no foothold and you have to use the wall like a foothold!
	**P6:** From the ground you learn how to evaluate the risks that a route entails and see that, over there, it’s risky and you could fall. And then too, for the same route, you can evaluate the difference between two ways of going forward (the one that’s going to take the most energy)… of course!!! that’s what the job of route finding is all about… What’s the way that’s going to cost me the least amount of energy and get me as far along as possible! That’s it: I try to record everything I’ll have to do in advance… all the effort I’m going to have to make in advance, right to the top! Doing and effort (two things): When I read, relying on my experience, I know how much energy I’ll have to use, bit by bit, from the start. If you don’t manage your energy, you won’t know when you should rest and how useful that is: Make the climb as easily as you can. Economize as much as you can. There’s obviously a link between reading the route and performance!
	**P10:** When I read a route, I try and map out the big traps… I especially look at the start, the start, ok… I imagine… the big traps and I tell myself “ok, you’ve got to get there right handed, if I don’t get there with my right I’m screwed. Next, I take everything apart and tell myself that to get there right-handed, I absolutely have to do this, this, this, this and this… watching out for the big traps like I said (a big trap is a place where if you get there with the wrong hand, you’ll fall… or you’re going to get tired, you’ll have to go back or you’ll have to change hands, whatever!). I really try to see that ‘for sure that grip is right-handed,” ok… then from below I’ll take it to my right then… I’ll do a re-launch there… here, there’s got to be a half-way spot and not a key grip… so, yeah, stuff like that’… and the feet follow… of course… and it’s true you think of your feet too, but generally speaking you concentrate more on the hands (the grips), but the move you’re going to make always depends on your feet!!! So I also watch the feet… if for instance I’ve got a vertical climb and I see a foothold is very far, it’ll be sure to be a drop knee or stuff like that… rest-spots on the route… I look for where I’ll be sure to be able to rest… like, on a corner…


## Discussion

To the best of our knowledge, the present study represents the first qualitative investigation of performance parameters as perceived by experts in predicting lead sport climbing performance. Precisely, the primary aim of the present study was to identify sport climbing performance predictors perceived as most influential in lead sport climbing and, secondly, to describe the role and function of route previewing in climbing performance. Our analysis revealed a wide-range of psychological, physiological, biomechanical and sociological climbing performance parameters that include passing cruxes, strength and conditioning aspects, interaction with the environment, possessing a good climbing movement repertoire, risk management (referring mainly to the risk of failing rather than the risk of injury), route management, mental balance, peer communication, and route preview. Route previewing emerged as the critical parameter that influenced cognitive and physical preparedness prior to ascent.

Participants in our study emphasized that performance output and success were, ultimately, determined by the interaction between different parameters that contributed to the configuration of the profile of the climber, ranging from individual such as anthropometrics profiles (Watts et al., 2003) to more external such as characteristics of the route (de Geus et al., 2006). Most technical decisions made by climbers on high grade climbing routes are automatically processed. Possessing a good climbing repertoire developed through training and “stress-tested” on increasingly more difficult standards of climbing routes would appear fundamental. Inherent in gaining a sound climbing repertoire would be the development of a high level of strength and conditioning.

In addition to the parameters more common to most competitive sporting disciplines in general, such as mental balance and risk management, participants in our study highlighted other more climbing-specific parameters such as peer communication and those related to route previewing (see further discussion below for the latter). Indeed, our participants reported that peer communication influenced climbers’ behaviors; it is still very common to see competitors route previewing together, and trying to find the best solutions to reach the top of a given route together despite being, actually, opponents. This, whilst today is considered to be a healthy form of rivalry, may well disappear in future once climbing reaches the standard competitive level more common to other more traditional competitive environments.

When it comes to route previewing, the views of the sample interviewed were that even if climbers were of equal biological (e.g., height) and physical capabilities (e.g., strength), route preview would make the difference between succeeding and failing on an ascent. Elite climbers are lean, muscular and likely able to climbing routes of a comparable standard. Therefore, given they are similar in that respect, route preview is likely the key parameter. Our participants declared that route preview allows individuals to mentally rehearse expected movement sequences in advance, identify and plan for crux sections thereby preserving energy and reducing the risk of non-completion due to a fall. Thus, route preview would allow the individual to reciprocally process the demands of the climbing route in regard of the key parameters highlighted to accurately appraise the challenges faced, and plan the best course of action required.

There is limited research on the role of psychological processing of climbing route information on climbing performance. Despite authorship teams suggesting that the ability to correctly visualize and interpret climbing route information prior to an ascent as an essential climbing skill (Boschker et al., 2002; MacLeod et al., 2007), and elite climbing competitors reporting a lack of climbing route knowledge as a handicap prior to performing in competition (Ferrand et al., 2006), the efficacy of route preview had not been tested experimentally until Sanchez et al. (2012). When examining the efficacy of pre-ascent climbing route visual inspection on indoor sport climbers, they found that expert climbers benefited most from route preview by making fewer stops and stops of a shorter duration during their ascent (Sanchez et al., 2012). Their findings went in line with intervention studies that have shown that techniques such as imagery and video-modeling do influence climbing performance positively (e.g., Sanchez and Dauby, 2009).

In fact, route previewing likely plays a significant role in climbing performance. Whereas competitors in other sports might see their opponents performing (e.g., in gymnastics, figure skating, trampoline) and practice on the itinerary where competition will take place (e.g., a rally driving route, a cycling circuit, a golf course), in sport climbing none of this is permitted. Thus, if a climber misjudges a sequence of holds that s/he has never done before (nor has ever seen anyone doing), s/he will likely fail/fall. In sports where environmental constraints change across competitions, specific preparation has been suggested as critical to successful performance (Eccles et al., 2009). Findings from the present study provide further qualitative information on how accurate route previewing is believed to function, optimizing the planning for the ascent in two distinct manners. Advanced and expert climbers conferred central value to route previewing, in line with recent work from Seifert et al. (2017), who suggested that this activity may help climbers to pick up functional information about reachable, graspable and usable holds to chain movements and find the way up on the wall. Such ability to correctly view and interpret climbing route information prior to an ascent was confirmed as an essential skill when it comes to prepare and plan ascents, both cognitively and physically. That is, the climber would estimate how the ascent is likely to be best achieved in relation to planning strategically the progression on the route (ascent strategy forecasting) and enhancing strategic management in relation to the effort exerted on the route (ascent effort forecasting). Our participants described how such planning for an ascent allows them to: select an accurate and comprehensive movement repertoire relative to the specific demands of the route and reject ineffective movement; optimize effective movements; and link different movements upward the entire route.

The present study provides a comprehensive view of parameters to consider when planning, designing and delivering holistic and coherent training programs aimed at enhancing climbing performance. In climbing, when performing on-sight, a mistake may result in failure to complete the route at that time point, or result in later failure due to increased fatigue in recovering from the original mistake. Despite a mistake the climber may still complete the ascent but at an increased physical cost (i.e., energy expenditure), which may have short-term implications for additional planned ascents, especially in competition. Additionally, in competition, a sport lead climbing mistake may also result in non-completion due to being timed out. Failing to complete an ascent also means for the climber that the given route will not be performed on-sight. If programs and training regimes are to be developed for climbers, the specific characteristics of the different climbing modalities (e.g., on-sight vs. pre-practiced) and the specific interacting parameters that influence performance need further consideration. For instance, and probably due to the consequence of a fall in competitive climbing, a clear distinction was made between on-sight and pre-practiced modalities.

When it comes to competition, previous research has shown that pre-performance psychological states influence subsequent performance (Sanchez et al., 2010). Other physiological and attentional changes due to a heightened stress have been shown in climbing populations. For instance, Pijpers et al. (2003) investigated anxiety and performance relationships in climbing and found anxiety was exhibited at three levels; subjective, physiological and behavioral, and that increased anxiety resulted in greater uncertainty in movement sequence and hold selection. In a later study, Pijpers et al. (2006) investigated anxiety in perceiving and realizing affordances and found anxiety narrowed the visual field and that the climber’s perception of the actions necessary to progress were altered by their emotional state. In both studies, Pijpers et al. (2003, 2006) show that controlled and fluid movements are vital for success in climbing. Studies that examine the relation between route previewing, emotions and actual climbing performance is warranted to gain further knowledge and understanding on how to prepare climbers best when seeking optimal climbing performance.

Overall, findings from the present study indicate that the combination of the abovementioned parameters is necessary to progress up the route in general, and to overcome particularly difficult sections (cruxes). However, to elucidate these parameters further, it is necessary to consider their inter-relatedness and frame these within the environmental context of elite sport climbing performance. As the sport of climbing continues to develop, our findings provide a basis for further research that shall examine further how, each of these performance parameters identified, can most effectively be enhanced and optimized to influence performance positively.

## Conclusion

The sport of climbing is rapidly developing whilst our understanding of the factors that influence performance is limited, and programs to enhance performance lack a robust ecologically valid evidence base. Our findings provide tentative for the design and delivery of training programs aimed at enhancing indoor sport climbing performance. In the present study, a comprehensive range of climbing performance parameters implicated in facilitating climbing performance achievement were identified. The importance of route previewing, which had been identified in previous studies, was confirmed. Current programs are typically developed on an *ad hoc* basis and, as a result, key performance parameters may be neglected. Even small differences in performance can be critical during elite competitions (*conf*. Sanchez et al., 2010), so a more holistic and coherent approach to training can be adopted now that a comprehensive range of predictors have been identified.

As the sport continues to develop, these new insights will also provide a basis for further investigation to establish how these key performance-related parameters can be most effectively enhanced. Taken together, findings from the present study provide a preliminary conceptual model with which to understand optimal lead-climbing performance on artificial climbing structures. Nevertheless, findings shall be taken with caution when it comes to generalize to the other two disciplines included in the Olympic program as they are very different in nature when compared to that of lead-climbing, even though competitors are requested to combine the three disciplines.

## Ethics Statement

This study was carried out in accordance with the recommendations of the Université catholique de Louvain, Faculty of Psychology and Education Sciences. All subjects gave written informed consent in accordance with the Declaration of Helsinki. The protocol was approved by the Post-Graduate Committee, Department of Emotion, Cognition and Health.

## Author Contributions

XS conceived and designed the study, carried out and transcribed the interviews, and wrote the first draft of the manuscript. XS and MT organized the database and performed the qualitative analysis. All authors contributed to manuscript developing and writing until manuscript was submitted in its final version.

## Conflict of Interest Statement

The authors declare that the research was conducted in the absence of any commercial or financial relationships that could be construed as a potential conflict of interest.
